# Single-Ring Intermediates Are Essential for Some Chaperonins

**DOI:** 10.3389/fmolb.2018.00042

**Published:** 2018-04-27

**Authors:** Jay M. Bhatt, Adrian S. Enriquez, Jinliang Wang, Humberto M. Rojo, Sudheer K. Molugu, Zacariah L. Hildenbrand, Ricardo A. Bernal

**Affiliations:** ^1^Department of Chemistry, The University of Texas at El Paso, El Paso, TX, United States; ^2^Department of Pharmacology, School of Medicine, Case Western Reserve University, Cleveland, OH, United States; ^3^Inform Environmental, Dallas, TX, United States

**Keywords:** chaperonins, GroEL, phiEL, HSP60, protein folding, single-ring chaperonins

## Abstract

Chaperonins are macromolecular complexes found throughout all kingdoms of life that assist unfolded proteins reach a biologically active state. Historically, chaperonins have been classified into two groups based on sequence, subunit structure, and the requirement for a co-chaperonin. Here, we present a brief review of chaperonins that can form double- and single-ring conformational intermediates in their protein-folding catalytic pathway. To date, the bacteriophage encoded chaperonins ϕ-EL and OBP, human mitochondrial chaperonin and most recently, the bacterial groEL/ES systems, have been reported to form single-ring intermediates as part of their normal protein-folding activity. These double-ring chaperonins separate into single-ring intermediates that have the ability to independently fold a protein. We discuss the structural and functional features along with the biological relevance of single-ring intermediates in cellular protein folding. Of special interest are the ϕ-EL and OBP chaperonins which demonstrate features of both group I and II chaperonins in addition to their ability to function via single-ring intermediates.

## Introduction

All the information required for macromolecules to acquire their correct three-dimensional structure and to undergo large conformational changes is found in the primary structure. Some proteins can refold on their own while others require assistance in regaining structural integrity and biological activity in the event of misfolding. Chaperonins are large complexes that are responsible for refolding these misfolded proteins. They constitute a highly conserved family of functionally and structurally related protein complexes that assist in the proper folding of non-native proteins involved in a wide variety of cellular processes (Brocchieri and Karlin, 2000; Henderson et al., 2010). In the absence of protein-folding assistance, cells accumulate misfolded protein and protein aggregates that eventually lead to cell death (Dekker et al., 2008; Sukhanova et al., 2012). Chaperonins are multi-subunit assemblies that form an internal protein-folding chamber that segregates misfolded substrate proteins from cytoplasmic constituents that can interfere with correct protein-folding. The general structure of chaperonins includes three separate domains that execute specific functions (Schoehn et al., 2000; Iizuka et al., 2004; Spiess et al., 2004). The apical domain is a highly flexible domain that interacts with the substrate protein and with a co-chaperonin that closes the opening to the protein-folding chamber after the substrate has entered (Saibil et al., 1993; Booth et al., 2008; Zhang et al., 2010). The intermediate domain acts as a hinge between the apical and the equatorial domain which is responsible for contacts between the two rings. The equatorial domain contains the nucleotide binding pocket and is responsible for conformational changes that drive the protein-folding cycle (Braig et al., 1994; Ditzel et al., 1998; Zhang et al., 2010).

Historically, chaperonins have been categorized into two groups according to their sequence similarity, the number of subunits and their arrangement, and their need for a co-chaperonin (Cheng et al., 1990; Reissmann et al., 2007; Techtmann and Robb, 2010; Lopez et al., 2015; An et al., 2017). Over the years, chaperonins with single-ring intermediates have been identified in eukaryotes and more recently in viruses (Horwich et al., 1993; Shaburova et al., 2006; Cornelissen et al., 2012; Hildenbrand and Bernal, 2012; Molugu et al., 2016; Semenyuk et al., 2016; An et al., 2017; Marine et al., 2017). Their protein-folding mechanisms, however, were poorly understood because they were largely based on knowledge obtained from studies of the bacterial groEL/ES chaperonin or its respective single-ring mutants (Sun et al., 2003; Liu et al., 2009; Kovács et al., 2010; Illingworth et al., 2011, 2015; Enriquez et al., 2017). Recent cryo-EM structural analyses on the ϕ-EL phage-encoded chaperonin revealed that it undergoes ring dissociation to form single-ring intermediates upon ATP hydrolysis (Hertveldt et al., 2005; Kurochkina et al., 2012; Semenyuk et al., 2015; Molugu et al., 2016). The novel single-ring intermediates of the ϕ-EL and OBP chaperonin are like those reported for the human mitochondrial chaperonin (Viitanen et al., 1992; Levy-Rimler et al., 2001). These single ring intermediates, along with other structural and functional features, differentiate ϕ-EL and OBP from commonly described group I and group II chaperonins.

## Characteristics of group I and II chaperonins

Group I chaperonins like the *Escherichia coli* chaperonin groEL and its co-chaperonin GroES (together denoted as groEL/ES), are typically found in eubacteria with the exception of the eukaryotic mitochondrial heat shock protein 60 (hsp60) and its co-chaperonin heat shock protein 10 (hsp60/10) (Fenton and Horwich, 1997). They are characterized as homo-tetradecamers composed of two stacked seven-membered rings (see Table 1) (Horwich et al., 2009; Enriquez et al., 2017). In addition, group I chaperonins possess a staggered (1:2) inter-ring subunit organization where one subunit in one ring directly contacts two subunits in the opposite ring (Braig et al., 1994; Ditzel et al., 1998; Hildenbrand and Bernal, 2012). The defining feature of group I chaperonins is that they require the assistance of an additional co-chaperonin protein that acts as a lid to isolate the central protein-folding chamber (Hayer-Hartl et al., 2016). In the absence of co-chaperonin, group I chaperonins can prevent non-native protein aggregation but are unable to fold them (Ellis, 2003; Horwich et al., 2009).

**Table 1:** Features of group I and II chaperonins compared to ϕ-EL.

	**Group I**	**Group II**	**ϕ-EL**
Source	Bacteria, *Homo sapiens*	Archaea and eukaryotes	Bacteriophage
Location	Cytoplasmic and endosymbiotic organelles	Cytoplasmic	Cytoplasmic
Substrate	**Promiscuous**	Substrate-specific	**Promiscuous**
Subunits per ring	7	7–9	7
Oligomeric organization	**Homo-oligomeric**	Hetero-oligomeric	**Homo-oligomeric**
Co-chaperonin	Required	**Not Required**	**Not Required**
Inter-ring Interactions	Out of register (1:2)	**In-register (1:1)**	**In-register (1:1)**
Ring separation	No	No	Yes

*Items in bold demonstrate similarities between ϕ-EL chaperonin and Group I and II chaperonins*.

Group II members include chaperonins from archaeal (Mm-cpn) and eukaryotic cells (TriC). These chaperonins can be homo or hetero-oligomers consisting of 7–9 subunits per ring (see Table 1). These group II complexes have an in-register (1:1) inter-ring subunit arrangement where each subunit contacts only one subunit in the opposite ring (Braig et al., 1994; Ditzel et al., 1998; Hildenbrand and Bernal, 2012). Group II chaperonins do not require a co-chaperonin for proper protein-folding due to an extra structural protrusion atop the apical domain that rearranges itself upon ATP hydrolysis to form a built-in lid that seals the central cavity (Ditzel et al., 1998; Kusmierczyk and Martin, 2003; Joachimiak et al., 2014).

## The ϕ-EL single-ring atpase cycle

Typically, bacteriophages will utilize the host chaperonin to process nascent viral polypeptides. However, the ϕ-EL chaperonin encoded by the *Pseudomonas aeruginosa* is unique in that it is the first of only two chaperonin groEL orthologs that have been identified in the phage genome (phage OBP being the other, see below). Chaperonin ϕ-EL possesses structural features of both group I and II (Molugu et al., 2016). The ϕ-EL chaperonin is like group I chaperonins in that it forms a homo-oligomeric tetradecameric complex and does not have substrate specificity. On the other hand, the similarities between ϕ-EL and group II chaperonins include the lack of a co-chaperonin and an in-register (1:1) subunit arrangement at the inter-ring interface (Table 1).

**Figure 1:**
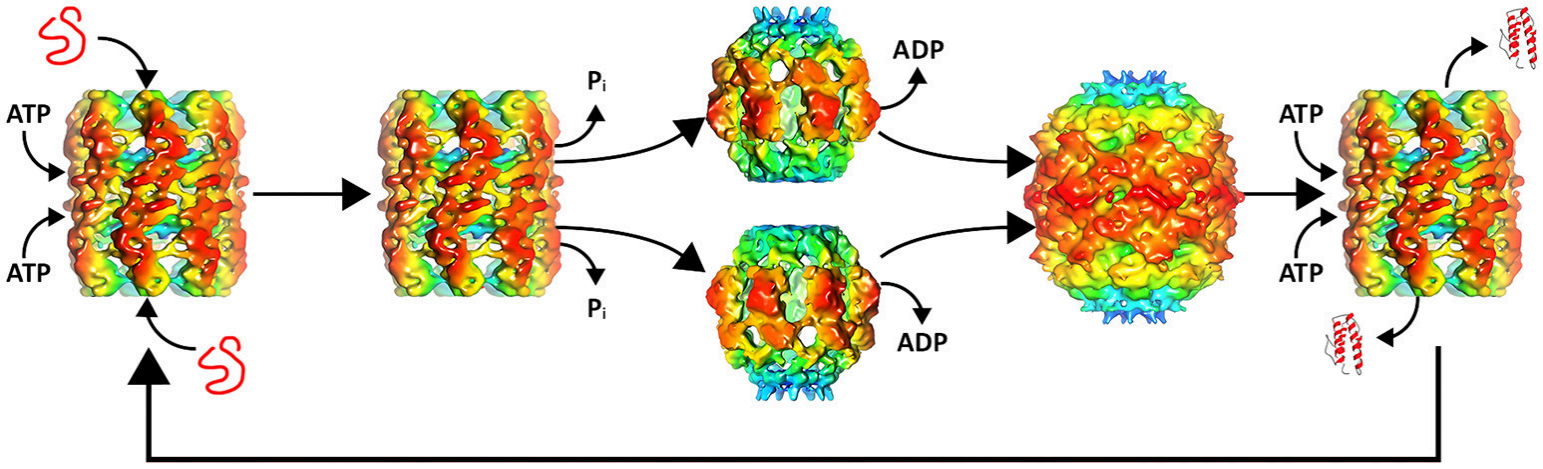
ϕ-EL protein-folding catalytic cycle. A misfolded substrate enters each of the two ATP bound chaperonin internal chambers. ATP hydrolysis induces apical domain rearrangement resulting in the closure of the internal chamber as well as ring separation. ADP removal allows the rings to reassemble to form the APO conformation. Renewed binding of ATP opens the protein-folding chamber allowing the folded substrate to exit and the cycle to begin again. Structures of intermediates were generated with chimera using the deposited maps EMD-6492, EMD-6493, and EMD-6494.

Recent electron microscope reconstructions have demonstrated that nucleotides control the conformational state of the chaperonin and that the substrate is the trigger that allows progression of the chaperonin along the catalytic protein-folding cycle (Molugu et al., 2016). In the absence of substrate and presence of ATP, the ϕ-EL chaperonin forms an open double-ring conformation that is primed for substrate binding (Figure 1). This conformation is stable until the substrate binds and triggers ATP hydrolysis. This *in-vitro* behavior makes sense because the chaperonin without substrate would deplete ATP reserves in futile hydrolysis reactions. ATP hydrolysis by both rings simultaneously triggers ring separation resulting in a more than two-fold enlargement in volume of the internal cavity (Figure 1). The enlarged protein-folding chamber enables the encapsulation of the 116 kDa denatured β-galactosidase protein, a substrate too large to be folded by groEL/ES (Ayling and Baneyx, 1996; Molugu et al., 2016). Interestingly, hydrolysis of ATP simultaneously triggers an extreme downward tilt of the equatorial domains that result in ring dissociation into two single-ring complexes (Molugu et al., 2016). This contributes in large part to the expansion of the internal chamber. At the apical end, ATP hydrolysis also induces closure of the internal chamber in what appears to be an iris-like rearrangement of the apical domains, circumventing the need for a co-chaperonin to act as a lid to create an isolated internal chamber. The two rings then re-associate to form a closed double-ring complex that then relies on the binding of ATP to release folded substrate and initiate another iteration of protein-folding (Molugu et al., 2016). The ϕ-EL chaperonin probably operates via a one-stroke protein-folding mechanism due to the simultaneous activity of both rings, as depicted in Figure 1. Furthermore, inter-ring negative cooperativity is likely eliminated since both rings appear to fold proteins simultaneously and therefore also must bind ATP to both rings (Molugu et al., 2016).

Most of what is known about single-ring chaperonins is largely based on studies of the ϕ-EL chaperonin and gro-EL single-ring mutant (SR1) (Weissman et al., 1995; Chen et al., 2006; Molugu et al., 2016). Cryo-EM structural analysis of the SR1-D398A groEL/ES revealed an 80% expansion of the volume of the central cavity compared to the expanded double-ring conformation (Chen et al., 2006). This expanded protein-folding cavity is also observed in ϕ-EL where it probably evolved to accommodate large viral proteins that cannot be encapsulated by the host double-ring chaperonin (Wolf, 2006; Molugu et al., 2016). Again, this was proven to be the case because the ϕ-EL chaperonin was able to effectively fold β-galactosidase, a protein that is not accommodated by groEL (Molugu et al., 2016).

## The OBP phage chaperonin

In addition to the ϕ-EL chaperonin, there is emerging evidence that many chaperonins may share the single-ring intermediate in their protein-folding catalytic cycle. Recently, another viral encoded chaperonin in the genome of *Pseudomonas* phage OBP has been purified as a single-ring complex (Semenyuk et al., 2016). The OBP gene product 246 (gp246) has been shown to form heptameric single-rings by electron microscopy. Although it was purified exclusively as a single-ring, this single-ring is likely an intermediate conformation in the protein-folding cycle of the OBP chaperonin. Like ϕ-EL, it does not require a co-chaperonin for its biological activity in *in-vitro* experiments (Semenyuk et al., 2016). All single-ring forming chaperonins studied to date form the single-ring as a conformational intermediate in the protein-folding cycle and do not function as a single-ring complex exclusively. It is anticipated that OBP gp246 will behave similarly and future X-ray structures or cryo-EM reconstructions will shed more light on the details about the OBP gp246 protein-folding cycle.

## The human mitochondrial Hsp60/10 protein-folding mechanism

Naturally occurring single-ring chaperonins like hsp60/10 were not well studied due to the instability of the functional complex *in-vitro* (Levy-Rimler et al., 2002; Vilasi et al., 2014). This lack of knowledge led researchers to make assumptions about single-ring chaperonins based on studies performed on groEL/ES single-ring mutants (Viitanen et al., 1998; Chen et al., 2006; Liu et al., 2009; Kovács et al., 2010). The human mitochondrial hsp60/10 chaperonin is the eukaryotic homolog of the bacterial groEL/ES complex and assists in maintaining the proper folding of newly imported and stress denatured mitochondrial proteins (Cheng et al., 1990; Horwich, 1990; Lubben et al., 1990; Dickson et al., 1994). Although the majority of hsp60 chaperonin resides in the mitochondrial matrix, numerous studies have now implicated its involvement in a variety of cellular processes at extra-mitochondrial locations (Singh et al., 1990; Soltys and Gupta, 1996, 1997, 1999; Itoh et al., 2002; Hildenbrand and Bernal, 2012; Henderson et al., 2013; Cappello et al., 2014). A functionally compromised hsp60/10 chaperonin complex in humans can lead to mitochondrial dysfunction and has been implicated in various neurodegenerative disorders (Magen et al., 2008; Parnas et al., 2009).

Early studies using chimeric groEL/hsp60 chaperonins revealed a single-ring hsp60 complex that retained the ability to fold target proteins *in-vitro* nearly identical to its wild-type counterpart (Nielsen and Cowan, 1998; Nielsen et al., 1999). This observation led to the conclusion that the mitochondrial single-ring chaperonin can maintain productive protein-folding without the use of double-ring complexes. Additionally, the expression of hsp60/10 proteins in an *E. coli* strain devoid of groEL/ES demonstrated that the hsp60/10 can compensate for the loss of groEL/ES (Nielsen et al., 1999).

More recent TEM and X-ray crystallographic investigations have provided strong evidence that the human mitochondrial chaperonin utilizes both double- and single-ring intermediates during its ATPase cycle (Levy-Rimler et al., 2001; Nisemblat et al., 2014, 2015; Vilasi et al., 2014; Enriquez et al., 2017). A mutant human hsp60 complexed with mouse hsp10 was crystallized resulting in a symmetric “American football” shaped structure (Hartman et al., 1992; Nisemblat et al., 2015). In addition, a 100° rotation of one subunit in each ring of the crystal structure indicated the intra-ring positive cooperativity observed in groEL is also not conserved in the mitochondrial chaperonin. Negative stain electron-microscopy on the nucleotide free wild-type human mitochondrial hsp60 complex revealed it forms a symmetrical, and stable tetradecameric complex that requires the presence of substrate to initiate ATPase activity (Enriquez et al., 2017). Negative stain electron microscope investigations of hsp60 have also demonstrated that it favors a tetradecameric complex in the presence of ATP, and a football complex in the presence of ATP and hsp10 (Levy-Rimler et al., 2001). It is still unclear whether ring-expansion occurs in the hsp60/10 single-ring complex and whether it allows for the folding of large proteins or if it simply doubles the protein-folding capacity when under stressful mitochondrial conditions.

The *in-vitro* analysis of the hsp60/10 ADP complex is difficult because biochemical studies indicate that hsp60 has an affinity for hsp10 that is so low in the presence of ADP that the affinity is nearly immeasurable (Nielsen and Cowan, 1998). Subsequent investigations demonstrated that the addition of ADP has little effect on the ATPase activity of hsp60/10 (Nielsen and Cowan, 1998; Levy-Rimler et al., 2001). Despite the evidence for hsp60/10 single-ring activity, the exact cellular conditions that coerce the formation of the hsp60/10 single-complex have yet to be elucidated (Viitanen et al., 1992; Nielsen et al., 1999; Nisemblat et al., 2015). Clearly, additional studies that include high resolution structural information of the single-ring intermediate are required to get a better understanding of how the hsp60/10 chaperonin folds a substrate protein.

## groEL/ES complex and single-ring intermediates

Recently, Yan et al. (2018) suggested that the groEL/ES complex may also be forming single-ring intermediates (Yan et al., 2018). This was observed in groEL mutants in the presence of the ATP analog ADP·BeFx which is supposed to mimic the ATP bound state, the ADP·Fx that mimics the transition state of ATP hydrolysis, and ADP·VO_4_ that mimics the post-hydrolysis state. ADP·BeFx binding to the *trans* ring of the asymmetric groEL/ES complex triggers ring separation. The separated rings reassemble after groES and ADP dissociate from the former *cis* ring. Preventing ring separation via mutagenesis led to complexes with reduced activity *in-vitro* and *in-vivo*. In our hands, these nucleotide analogs yielded off-pathway intermediates suggesting that the analogs were not behaving as predicted compared to the natural nucleotides (ATP and ADP) (unpublished data). The absence of a substrate prevented progression of the chaperonin to the next conformational intermediate and so we decided to simply use the natural nucleotides to avoid structural artifacts.

## Conclusion

Single-ring intermediates have been identified for ϕ-EL, OBP and hsp60/10 chaperonin complexes. Recently, groEL/ES complexes have also been suggested to operate via single-ring intermediates although further data is required to prove that single-rings are relevant. The naturally occurring single-ring intermediates are an integral part of bacteriophage and human mitochondrial chaperonin protein-folding catalytic pathways. The exact sequence, structural and cellular conditions that regulate the formation of these single-ring intermediates in still unknown. Further insight into single-ring chaperonins is important since the human hsp60 is now implicated in the onset of a wide variety of diseases including arthritis, cancer, and neurodegenerative disorders (Hansen et al., 2002; Parnas et al., 2009; Ghosh et al., 2010; Campanella et al., 2012; Henderson and Martin, 2013).

## Author contributions

JB, AE, ZH, JW, and RB: contributed to the writing of the manuscript; JW and SM: performed all of the bioinformatics calculations and generated Figure 1; HR: generated Figure 1. All of the authors contributed to the editing of various versions of the manuscript and all read the final version.

### Conflict of interest statement

ZH is employed by the company Inform Environmental, LLC. The other authors declare that the research was conducted in the absence of any commercial or financial relationships that could be construed as a potential conflict of interest.
